# A nationally representative dataset of 1,549 Americans aged 18 to 94 on interest in, experience with, and barriers to cogeneration, defined as working with older and younger people for social good

**DOI:** 10.1016/j.dib.2022.108753

**Published:** 2022-11-17

**Authors:** Cal J. Halvorsen, Bruce Kelley, Jim Emerman, Stefanie Weiss, David Gleicher, Jacob Stolmeier, Mark Lush

**Affiliations:** a Boston College School of Social Work, 140 Commonwealth Ave, Chestnut Hill, MA, USA; b Consultant, Lake Placid, NY, USA; c CoGenerate, San Francisco, CA, USA; d NORC at the University of Chicago, Chicago, IL, USA

**Keywords:** National and community service, Cogenerational service, Age diversity, Generation Z, Millennials, Generation X, Baby boomers, Silent generation

## Abstract

This dataset focuses on Americans’ interest in, experience with, and perceived barriers to working with members of other generations to improve the world around them. It includes responses from a March 2022 survey of 1,549 people between the ages of 18 and 94 who lived in the U.S. using the NORC at the University of Chicago AmeriSpeak® Panel. To increase the representativeness of the sample, the survey was offered both online and by phone. The sample is drawn from a probability-based panel designed to be representative of the U.S. household population. Questions focused on respondents’ efforts (paid or volunteer) to improve the world around them, with a particular focus on cogenerational work with people at least 25 years older and younger than themselves. Respondents answered questions about their interest in and experience with cogenerational work as well as perceived barriers to it. Respondents were also asked to identify specific issues that they would like to work on with people of different generations (e.g., mental health, education, environment), their beliefs on if and how younger and older people working together might reduce divisions in society, and their engagement with people of different generations outside of their families. The complete dataset with 189 variables (10 of which are string/text variables from open-ended responses) is available both as a Stata .do file as well as in two .csv files. Two codebooks (one simplified, one full) and a project report from NORC that details the dataset's weighting and other methodological information are also available. This point-in-time dataset can be used for univariate, bivariate, and multivariate analysis and may be useful to researchers, social sector leaders, and policymakers interested in multigenerational efforts to solve social problems.

**Specifications Table**

**Table:** 

Subject	Social Sciences / Sociology
Specific subject area	Cogenerational work for the social good (defined as paid work, volunteering, and other ways people engage across generations on issues they care about). Similar terms: multigenerational, intergenerational.
Type of data	.csv file (dataset with numbers) .csv file (dataset with labels) .do file (Stata dataset) .pdf file (codebook, simplified) .pdf file (codebook, full) .pdf file (NORC project report)
How the data were acquired	Data were acquired in English from NORC at the University of Chicago's AmeriSpeak Panel using a custom questionnaire by this manuscript's authors.
Data format	Filtered data in both .csv (separate files for numbers and labels) and .do formats. (Note: Raw data were filtered to remove 92 responses for data quality reasons, described in this article.)
Description of data collection	The survey was fielded from March 9 to 23, 2022 by phone and online, depending on respondent's preference. The median survey time was 8 minutes. A general population sample of U.S. adults aged 18 and older was selected using the AmeriSpeak Panel. Sampling strata for this panel study were based on age, gender, race, Hispanic ethnicity, education, and other factors (a total of 48 sampling strata).
Data source location	Respondents within the AmeriSpeak Panel, who live throughout the U.S. and within each of the U.S. Census Bureau's four regions and nine divisions, took this survey.
Data accessibility	Repository name: Boston College Dataverse All data can be accessed at the following link: https://doi.org/10.7910/DVN/AIQ87J This archive is supported by the Boston College University Libraries and hosted by the Harvard Dataverse Network.

**Value of the Data**

• These data are useful in understanding the attitudes and experiences of U.S.-based adults on working with people of different ages to address social problems and improve the world around them.
• Researchers, social and public sector leaders, program managers, and policymakers may benefit from this dataset through its opinion- and experience-based questions and set of sociodemographic variables.
• These data can be analyzed with univariate, bivariate, and multivariate methods.
• The 1,549 respondents to this survey are a part of the NORC at the University of Chicago AmeriSpeak Panel, which is a probability-based panel designed to be representative of the U.S. household population.

## 1 Objective

The primary objective for creating this dataset was to better understand Americans’ interest in, experience with, and perceived barriers to working with members of other generations to improve the world around them, in addition to examining respondents’ general civic engagement and experiences with people of different generations. The secondary objective was to estimate population-level statistics relating to these topics using a nationally representative sample. This data article adds value to the report released soon after the creation of this dataset [1] in several ways. First, the dataset includes detailed information on each respondent. Second, it includes multiple variables not analyzed in the report published by CoGenerate (formerly Encore.org). And third, researchers will be able to use this dataset to conduct a variety of statistical analyses, whereas the report focused primarily on descriptive statistics and population averages.

## 2 Data Description

Two codebooks are available as supplemental files through the Boston College Dataverse [2]. The first, *Codebook_Simplified*, includes the survey questions, response options, and branching logic that were used for more than 95% of the respondents who took the survey online via a computer, smartphone, or tablet. The second, *Codebook_Full*, includes the same survey questions and response options for the online version (described as “CAWI” for computer-assisted web interviewing) as well as those for the phone interview (described as “CATI” for computer-assisted telephone interviewing). The questions and answer options sometimes changed so that, for example, pronouns switched from “I” in the CAWI version to “you” in the CATI version. For most purposes, the simplified codebook is enough to understand the survey questions.

The survey was designed to understand respondents’ general civic engagement (Q1-Q2 in the supplemental codebooks), their civically engaged work with people of different generations (Q3-Q10), their interest and intentions in moving into civically engaged work with people of different generations in the future (Q11-Q13, Q15-Q16), barriers to moving into this work (Q14), issues on which respondents might like to work with people of different generations (Q17-Q18), and general questions about the value of older and younger people working together and interacting more (Q19-29). Descriptive analysis of many, but not all, of the survey's findings can be found in the *Cogeneration* report [1].

Table 1 provides the characteristics of the dataset's sample using weighted and unweighted frequencies. The variables in Table 1 were not a part of our specific survey but were answered by respondents when they entered into the AmeriSpeak Panel with periodic updates by NORC. The sample is almost evenly distributed by gender with respondents from across the adult lifespan. Nearly two-thirds of the sample identified as White, non-Hispanic, with about one in six identifying as Hispanic and one in nine identifying as Black, non-Hispanic. Additional sample characteristics include educational attainment, marital status, employment status, 9-category household income, 9-category U.S. Census division, whether or not the respondent lives in a metropolitan area as defined by the U.S. Office of Management and Budget as a core-based statistical area, housing type, and number of people living in the household. Sample characteristic variables available in the dataset but not shown in Table 1 include survey start and end dates, survey duration (in minutes), survey mode (web vs. phone interview), continuous and 7-category age, 4- and 18-category household income, state, 4-category U.S. Census Bureau region, household internet access, home type, phone service and usage (e.g., landline, cellphone), and number of household members by specific age groups (i.e., 0-1, 2-5, 6-12, 13-17, and 18+ years). Unique identification number (CaseID) and weights are also provided.

**Table 1:** Sample Characteristics (*N*=1,549).

	Unweighted	Weighted
**Gender**		
Male	47.97%	48.49%
Female	52.03%	51.51%
**Age**		
18-29	14.46%	20.29%
30-44	26.60%	25.75%
45-59	23.31%	23.60%
60+	35.64%	30.36%
**Race/ethnicity**		
White, non-Hispanic	65.72%	62.54%
Black, non-Hispanic	11.43%	11.98%
Asian, non-Hispanic	3.10%	6.04%
Another race, non-Hispanic	1.48%	0.92%
2+ races, non-Hispanic	2.58%	1.64%
Hispanic	15.69%	16.87%
**Education**		
< High school diploma	4.65%	9.60%
High school graduate or equivalent	17.43%	28.31%
Vocational, technical school, some college, associate's degree	40.09%	27.07%
Bachelor's degree	21.43%	18.18%
Post graduate study, professional degree	16.40%	16.83%
**Marital status**		
Married	50.48%	49.80%
Widowed	4.26%	3.04%
Divorced	11.30%	9.78%
Separated	4.13%	4.18%
Never married	23.95%	27.52%
Living with partner	5.87%	5.68%
**Employment status**		
Working (paid employee)	51.45%	50.89%
Working (self-employed)	6.97%	7.74%
Not working (temporarily laid off)	2.19%	1.96%
Not working (looking for work)	3.62%	4.65%
Not working (retired)	22.85%	19.75%
Not working (disabled)	6.52%	7.06%
Not working (other)	6.39%	7.95%
**Household income**		
< $10,000	4.58%	4.70%
$10,000 to < $20,000	8.59%	8.76%
$20,000 to < $30,000	10.26%	10.03%
$30,000 to < $40,000	8.72%	7.60%
$40,000 to < $50,000	9.88%	9.05%
$50,000 to < $75,000	19.43%	19.06%
$75,000 to < $100,000	13.94%	12.80%
$100,000 to < $150,000	15.69%	17.47%
$150,000+	8.91%	10.52%
**U.S. Census division**		
New England	5.23%	4.68%
Middle Atlantic	10.52%	12.51%
East North Central	18.66%	14.23%
West North Central	9.94%	6.40%
South Atlantic	19.17%	20.46%
East South Central	5.42%	5.86%
West South Central	7.94%	11.95%
Mountain	8.97%	7.63%
Pacific	14.14%	16.29%
**Metropolitan area**		
Metro area	84.64%	84.45%
Non-metro area	15.36%	15.55%
**Housing type**		
Owned	66.95%	70.84%
Rented	30.34%	26.90%
Occupied without payment	2.71%	2.26%
**Household size**^a^		
1	18.01%	15.99%
2	35.51%	33.21%
3	15.88%	15.92%
4	13.04%	13.22%
5	9.68%	11.21%
6+	7.88%	10.46%
**Device**		
Computer^b^	40.41%	37.43%
Smartphone	53.65%	56.80%
Tablet	1.23%	1.03%
Phone interview	4.52%	4.49%

a The number of people who live in the household for at least three months per year, including the respondent.

b Includes desktop and laptop computers.

Table 2 shows the descriptive statistics for the weight variable. The mean weight is 1.00 and the median is 0.783. The methods for creating the weight variable are described in the next section.

**Table 2:** Weight descriptive statistics.

Mean (SD)	1.000 (0.840)
Smallest	0.027
25^th^ percentile	0.428
Median	0.783
75^th^ percentile	1.295
Largest	7.886

Lastly, three databases are available as supplemental files in the Boston College Dataverse [2]. Two are in comma separated value (.csv) format easily read by Microsoft Excel and other statistical software: one with numbers only (e.g., “0” and “1”) and one with labels (e.g., “no” and “yes”). The third is in Stata's .do format, complete with numbers and labels.

## 3 Experimental Design, Materials and Methods

CoGenerate, in consultation with the first and second authors, contracted with NORC to design and field this survey through the AmeriSpeak Panel.

The AmeriSpeak Panel has been described extensively in publications by NORC [3,4] and used by university-affiliated researchers for matters of public concern [5,6]. Designed to be representative of the U.S. household population, the panel consists, in 2022, of more than 54,000 members aged 13 and older within 43,000 households [7].

To create its sample, NORC used a multistage probability sample to represent the U.S. household population that included a selection of primary sampling units that are a county or group of counties that have a minimum population of 10,000. Within these primary sampling units, NORC then selected both urban and rural secondary sampling units from U.S. Census tracts or block groups. Within these secondary sampling units, NORC then created a list of nearly 3 million household units using U.S. Postal Service and in-field information that is estimated to provide more than 97% coverage of the U.S. household population. NORC researchers then randomly selected households from this list and recruited them into the panel by mail, telephone, and face-to-face interviews, with sampling strata based on race, ethnicity, and age. Those who join were offered modest incentives. The cumulative weighted household response rate for the AmeriSpeak sample is 21.9%. More details about the panel sample frame, sample selection, recruitment procedures, response rates, panel management and maintenance, and additional technical information is described on the AmeriSpeak website [7].

To increase transparency, we have made available the project report that NORC submitted to CoGenerate upon the completion of the survey (see *NORC_Project_Report* in the Boston College Dataverse[2]). This report provides a detailed overview of the survey's sampling strategies, pilot testing and fielding, panel and survey sample performance, data processing and quality review procedures, and statistical weighting. Much of this information aligns with the requirements of the American Association for Public Opinion Research's Transparency Initiative [8], of which NORC is a charter member. The remaining content in this section is derived from this detailed project report.

A total of 5,837 panelists were invited to take this survey. Of those, 1,549 (26.5%) completed the survey. For respondents who prefer to take surveys online, they received an initial email invitation on March 9, 2022 with five subsequent reminders through March 22. For respondents who prefer to take telephone surveys, they received phone calls between March 14 and 22, 2022. An incentive equivalent to $2 was offered to respondents for completing this study through a rewards program specific to the AmeriSpeak Panel.

The final sample size of 1,549 respondents excludes 92 cases that were removed for data quality concerns. Fifteen cases were removed due to speeding (i.e., respondents who completed the survey in less than one-third the median response time) and 90 were removed due to high refusal rates (i.e., respondents who skipped or refused more than half of their eligible questions). Thirteen of these cases were flagged in both categories. (Note: In one open-ended answer, a respondent provided information that could increase the chances of them being identified. As such, we deleted a portion of their answer to protect their identity. This edit does not greatly affect the interpretation of their answer.)

Due to how AmeriSpeak surveys are programmed and panelists are invited, respondents are not able to take the survey more than once and each respondent is identifiable based on a unique ID. As such, the quality of the responses is high, as the AmeriSpeak Panel does not suffer from issues relating to bots, fake profiles, respondents who were not invited to take the survey, nor people from within the same household repeatedly taking the same survey.

The sampling weights were developed by NORC to increase the representativeness of the sample to the U.S. household population. The final weight variable included in this dataset is a product of three weights: (1) the AmeriSpeak Panel weights that account for the probability of selection into the sample, recruitment nonresponse adjustments, and poststratification adjustments to match population benchmarks that includes age, gender, U.S. Census division, education, race and ethnicity, housing type, and household phone status; (2) specific base weights to this study to account for respondents’ selection probabilities, which are a product of the AmeriSpeak Panel weights and the inverse of selection probabilities associated with sample selection from the panel; and (3) specific final weights that are developed for all completed cases for this study that includes raking adjustment to align with population benchmarks, including age, gender, U.S. Census division, race and ethnicity, and interactions between age and gender, age and race/ethnicity, and race/ethnicity and gender.

A publicly available report that focused primarily on descriptive statistics and population averages was published soon after the creation of this dataset, in June 2022 [1].

## Ethics Statements

NORC operates its own Institutional Review Board, which is registered with the U.S. Department of Health and Human Services Office for Human Research Protections and has obtained a Federal Wide Assurance (#FWA00000142). The NORC IRB, which reviews all research using the AmeriSpeak Panel, reviewed this study protocol (Protocol ID: 22-02-662). Respondents all provided informed consent to join the AmeriSpeak Panel and must agree to take part in each survey before receiving the questions.

## CRediT authorship contribution statement

**Cal J. Halvorsen:** Conceptualization, Formal analysis, Resources, Writing – original draft, Supervision. **Bruce Kelley:** Conceptualization, Methodology. **Jim Emerman:** Conceptualization, Methodology. **Stefanie Weiss:** Project administration, Writing – review & editing. **David Gleicher:** Formal analysis, Investigation, Data curation, Resources, Writing – original draft. **Jacob Stolmeier:** Supervision, Project administration, Resources. **Mark Lush:** Methodology, Resources, Writing – original draft.

## Declaration of Competing Interest

The authors declare that they have no known competing financial interests or personal relationships that could have appeared to influence the work reported in this paper.

## Data Availability

Cogeneration: A nationally representative survey of 1,549 Americans aged 18 to 94 on interest in, experience with, and barriers to working with older and younger people for social good (Original data) (Dataverse).
